# Population-level impact of ‘The Real Cost’ campaign on youth smoking risk perceptions and curiosity, United Sates 2018–2020

**DOI:** 10.18332/tid/174900

**Published:** 2023-12-12

**Authors:** Shaikha Aldukhail, Abdulaziz Alabdulkarim, Israel T. Agaku

**Affiliations:** 1 Department of Preventive Dental Sciences, College of Dentistry, Princess Nourah bint Abdulrahman University, Riyadh, Saudi Arabia; 2 Plastic Surgery, Department of Surgery, College of Medicine, Prince Sattam bin Abdulaziz University, Al Kharj, Saudi Arabia; 3 Division of Oral Epidemiology and Dental Public Health, University of California San Francisco, San Francisco, United States

**Keywords:** tobacco, adolescents, cigarette smoking, priority/special populations, mass media campaign

## Abstract

**INTRODUCTION:**

The FDA’s ‘The Real Cost’ tobacco prevention campaign aimed to counter tobacco marketing efforts directed toward children and youths. Our objectives were to explore the associations between exposure to the FDA’s campaign and cigarette risk perception among the US adolescent population, and between exposure and cigarette smoking curiosity among adolescents who never smoked cigarettes.

**METHODS:**

We analyzed 3 cycles of National Youth Tobacco Survey (NYTS 2018–2020, n=53738). Multivariable logistic regression models were fitted to measure the relationship between campaign exposure and cigarettes risk perception (among all), as well as the relationship between campaign exposure and cigarette curiosity (among cigarette never smokers).

**RESULTS:**

Majority of youths have reported exposure to the campaign 63% between 2018–2020. The odds of youths perceiving cigarettes as risky were 1.6 times higher among exposed compared to those not exposed (adjusted odds ratio, AOR=1.60; 95% CI: 1.43–1.79). There were some racial disparities in risk perceptions among Hispanics and Non-Hispanic Blacks across exposure groups. Exposure was associated with higher cigarettes curiosity odds among Hispanic youths who never smoked (AOR=1.26; 95% CI: 1.10–1.44) compared to their Non-Hispanic White peers.

**CONCLUSIONS:**

The FDA’s ‘The Real Cost’ campaign had exposure levels deemed essential for population-level perceptions change. Exposure was associated with youths having higher risk perceptions about the negative health outcomes related to cigarette smoking. However, students that never smoked were more curious about smoking with campaign exposure. Therefore, future health communication plans should consider both the potential benefits and possible unintended consequences prior to launching such campaigns.

## INTRODUCTION

Cigarette smoking continues to be a leading cause of preventable morbidity and mortality in the United States^1^. Tobacco use habits among most adults were established during their adolescent years. About 90% of current adult smokers initiated their smoking habit before they were 18 years old^1^. Further, tobacco smoking is a behavior sustained by addiction to nicotine, and, in a study, new smokers were found to exhibit symptoms of nicotine dependence within a few days of cigarette smoking initiation^2^. Historically, the tobacco industry has aggressively targeted adolescents in their marketing strategies^3^, identifying them as ‘replacement smokers’ and/or ‘learners’ in industry documents^4^. In this direction, the industry’s marketing has had an established role in youth tobacco use initiation; considering that marketing elevates curiosity levels, which may lead those who ‘never smoked’ to become susceptible to smoking, and thereafter increase their probability of tobacco experimentation and subsequent established use^5,6^. Consequently, developing effective interventions that aim to counter the industry’s marketing and prevent adolescents from initiating tobacco use remains a major public health priority.

Mass media campaigns are widely used to expose large numbers of a population to targeted health messages, making them an effective tool to reach and influence change in knowledge, attitudes, and health-related behaviors^7,8^. In 2009, the US Food and Drug Administration (FDA) gained regulatory authority over tobacco products through the Family Smoking Prevention and Tobacco Control Act, granting the FDA responsibility to educate the public about the adverse health effects of tobacco use, and prompting the launch of the national, youth-targeted, tobacco counter-marketing campaign ‘The Real Cost’.

‘The Real Cost’ campaign was developed based on the Theory of Planned Behavior, which hypothesizes that ‘change in behavior was a result of changes in beliefs that, in turn, influence attitudes toward a behavior, perceptions of associated social norms, and/or self-efficacy to engage in or refrain from a behavior’^8^. Thus, ‘The Real Cost’ was developed to influence youths’ tobacco use habits through raising negative attitudes and perceptions, shaping social and normative beliefs, and reducing influences of peer pressures^9-12^.

The message development strategy for young audiences was to focus on three youth smoking-related themes: loss of control and independence due to addiction, negative health consequences due to smoking (including cosmetic effects), and dangerous chemicals in cigarettes^9,13^. The overarching campaign message goal was to highlight how tobacco use leads to adverse health effects, which may be expressed in the scope of risk perceptions^12^.

Although youth-specific campaigns such as ‘The Real Cost’ have been implemented since 2014, extant research on the issue is from small, local studies that may have limited generalizability. To address this knowledge gap, the objectives of this study were to explore the association between exposure to campaign and cigarette risk perception among the US adolescent population, and the association between exposure and cigarette smoking curiosity among US adolescents who never smoked cigarettes.

## METHODS

### Study population, design, and setting

We analyzed 3 cycles of National Youth Tobacco Survey (NYTS 2018–2020, n=53738). NYTS is a nationally representative, annual, school-based, self-administered survey of cross-sectional samples of US private and public-school students in grades 6–12. It is used to assess self-reported tobacco-related beliefs, attitudes, behaviors, and risk factors. A total 53738 middle and high school students’ questionnaires were completed between 2018 and 2020; for more details about the sampling techniques, see the NYTS: Methodology reports^14-16^.

### Study variables

*Independent variable: Exposure to ‘The Real Cost’ campaign*

We assessed exposure to the ‘The Real Cost’ campaign with the question: ‘In the past 12 months, have you seen or heard about “The Real Cost” on television, the internet, social media, or radio as part of ads about tobacco?’. Response options were: yes, no, and not sure. We categorized respondents who answered yes as exposed. Responses of ‘not sure’ were classified as indeterminate.

*Dependent variables: Risk perception, and smoking curiosity*

The primary outcome in the study was self-reported ‘Cigarette smoking risk perception’ assessed with the question: ‘How much do you think people harm themselves when they smoke cigarettes some days but not every day?’. We regarded responses of ‘no harm’ and ‘little harm’ as negative, and those of ‘some harm’ or ‘a lot of harm’ as positive. We also assessed smoking curiosity among never smokers. ‘Curiosity towards cigarette smoking’ was assessed by the question: ‘Have you been curious about smoking cigarettes?’. Responses were dichotomized into ‘ definitely not’, and any other response (i.e. ‘probably not’, ‘probably yes’, and ‘definitely yes’), as this separated committed never smokers from susceptible never smokers^17^.

### Measures of tobacco use and exposure

Cigarette smoking status and other tobacco use behaviors were separated into groups: Established cigarette smokers (smokers who smoked at least 100 cigarettes), experimental cigarette smokers (ever smoked ≥1 puff but not yet smoked 100 cigarettes), and never cigarette smokers (not even one or two puffs). Non-cigarette tobacco product use was assessed by combining any positive response to the questions: ‘Have you ever tried smoking cigars, cigarillos, or little cigar, even one or two puffs?’, ‘Have you ever used chewing tobacco, snuff, or dip, even just a small amount?’, ‘Have you ever tried smoking tobacco in a hookah or waterpipe, even one or two puffs?’. In 2019 and 2020, a new question was included: ‘Have you ever tried a “heated tobacco product”, even just one time?’.

Living with a tobacco user was measured by the question: ‘Does anyone who lives with you now: ‘smoke cigarettes’; ‘smoke cigars, cigarillos, or little cigars’; ‘use chewing tobacco, snuff, or dip’; ‘use e-cigarettes (electronic cigarettes)’; ‘smoke tobacco in a hookah or water pipe’; ‘smoke pipes filled with tobacco (not water pipe)’; ‘use snus’; ‘use dissolvable tobacco products’; ‘smoke bidis (small brown cigarettes wrapped in a leaf)’; ‘heated tobacco product’, and ‘no one who lives with me now uses any form of tobacco’. Living with a tobacco user was measured with any response other than ‘no one who lives with me now uses any form of tobacco’. Other sociodemographic characteristics such as sex (male, female), race (Non-Hispanic White, Non-Hispanic Black, Non-Hispanic Asian, Hispanic, other), and school level (middle school or high school) were also included in the analyses.

### Statistical analysis

Descriptive statistics of frequency and percentages/prevalence with standard deviation (SD), were used to summarize the sociodemographic characteristics and categorical variables including campaign exposure and tobacco use measures. All percentages were weighted to yield nationally representative results, and to factor for the complex survey design. Multivariable logistic regression models were fitted to measure the relationship between campaign exposure and cigarette risk perception (among all participants), as well as the relationship between campaign exposure and cigarette curiosity (among cigarette never smokers). The reported adjusted odds ratios (AORs) and 95% confidence intervals (95% CIs) were adjusted for relevant demographic covariates; we also controlled for other non-cigarette tobacco product use, and household tobacco use.

All statistical analyses were performed using STATA version 14 (Stata Corp), and statistical significance was set at p<0.05.

## RESULTS

### Exposure to ‘The Real Cost’ messages

Between 2018 and 2020, the estimated exposure to the FDA’s ‘The Real Cost’ anti-smoking advertisements was 63% among adolescents in the US. The prevalence of exposure was 67% among Non-Hispanic Whites, 59% Hispanics, and 57% Non-Hispanic Blacks. Exposure was higher among those who were in high school (67%) and among males (65%). Exposure was higher among adolescents who smoked (68%), used other tobacco products (67%) or lived with family that used tobacco products (67%) (Table 1).

**Table 1:** Exposure to ‘The Real Cost’ anti-smoking campaign among adolescents aged 11–18 years, by sociodemographic characteristics, NYTS 2018–2020

*Characteristics*	*Weighted US population*		Exposure to campaign*	
	*n*	*% (SE)*	*n*	*% (SE)*
**Overall**	53738	NA	32244	62.70 (0.6)
**Sex**				
Female	26358	48.94 (0.4)	15556	60.84 (0.6)
Male	27025	51.06 (0.4)	16518	64.52 (0.7)
**Race/ethnicity**				
Non-Hispanic White	26212	55.20 (1.3)	17171	67.20 (0.7)
Non-Hispanic Black	6583	12.88 (0.8)	3554	57.05 (1.0)
Hispanic	15680	25.45 (1.0)	8826	58.62 (1.0)
Non-Hispanic Asian	2529	4.93 (0.5)	1366	55.24 (2.2)
Non-Hispanic Other	994	1.54 (0.1)	566	63.24 (2.3)
**School grade**				
Middle school	24934	43.98 (1.5)	13840	57.07 (0.7)
High school	28541	56.02 (1.5)	18288	67.17 (0.8)
**Cigarette smoking**				
Ever smoked^a^	8161	15.12 (0.6)	5182	67.83 (1.0)
Never smoked	45166	84.88 (0.6)	26933	61.91 (0.6)
**Other tobacco products use**				
Ever used	13134	24.49 (0.9)	8370	66.71 (0.8)
Never used	39774	75.51 (0.9)	23637	61.53 (0.7)
**Family tobacco use**				
Use tobacco	19229	37.47 (0.7)	12532	67.03 (0.6)
Does not use tobacco	32044	62.53 (0.7)	18906	60.80 (0.7)

a Ever smoked: has smoked ≥1 puff of a cigarette. All variables are reported with unweighted frequency and weighted percentage to account for complex survey design. SE: standard error. (%): weighted exposure prevalence.

* Results represent the number and weighted percentage of adolescents who replied ‘Yes’ to the following question: ‘In the past 12 months have you seen or heard “The Real Cost” on television, the internet, social media or radio, as part of ads about tobacco?’.

### Cigarettes risk perception

The odds of youths perceiving cigarettes as harmful or risky were 1.6 times higher in those who were exposed to ‘The Real Cost’ (AOR=1.60; 95% CI: 1.43–1.79) compared to not exposed. Hispanic adolescents who were exposed to the campaign were 30% less likely to have negative risk perceptions toward cigarettes (AOR=0.70; 95% CI: 0.60–0.80), compared to Non-Hispanic Whites. Similarly, among the not exposed, Hispanic adolescents were 40% less likely to report risk perceptions (AOR=0.60; 95% CI: 0.50–0.72), compared to not exposed Non-Hispanic Whites. Furthermore, compared to adolescents who never smoked, cigarette smokers who were expose to the campaign messages reported lower ‘risk perceptions’ (AOR=0.59; 95% CI: 0.52–0.68) and (AOR=0.27; 95% CI: 0.21–0.35), respectively, for experimental and established smokers. Not-exposed youths who were established smokers were 84% less likely to perceive cigarettes as risky (AOR=0.16; 95% CI: 0.11–0.25), compared to cigarette never smokers. Additionally, students who were exposed and were using other tobacco products were also 61% less likely to report cigarettes as risky (AOR=0.39; 95% CI: 0.34–0.45) compared to those who did not use any other tobacco products. Similarly, exposed adolescents who lived with family that used tobacco products were 28% less likely to perceive cigarettes as risky (AOR=0.72; 95% CI: 0.64–0.80) compared to those with non-tobacco using families (Table 2).

**Table 2:** Logistic regression analysis of the factors associated with exposure to ‘The Real Cost’ anti-smoking campaign and cigarettes risk perception among US adolescents aged 11–18 years, NYTS 2018–2020

*Covariates*	*Exposed*		*Not exposed*		*Not Sure*	
	*AOR (95% CI)*	*p*	*AOR (95% CI)*	*p*	*AOR (95% CI)*	*p*
**Sex**						
Female (Ref.)	1		1		1	
Male	0.69 (0.61–0.77)	**<0.0001**	0.80 (0.68–0.95)	**0.011**	0.71 (0.58–0.86)	**0.001**
**Race/ethnicity**						
Non-Hispanic White (Ref.)	1		1		1	
Non-Hispanic Black	0.84 (0.70–1.02)	0.081	0.75 (0.57–0.99)	**0.048**	0.83 (0.61–1.13)	0.238
Hispanic	0.70 (0.60–0.80)	**<0.0001**	0.60 (0.50–0.72)	**<0.0001**	0.65 (0.52–0.82)	**<0.0001**
Non-Hispanic Asian	0.99 (0.70–1.41)	0.085	1.36 (0.85–2.18)	0.197	0.91 (0.51–1.64)	0.764
Non-Hispanic Other	0.84 (0.55–1.27)	0.398	0.80 (0.44–1.44)	0.454	0.70 (0.36–1.36)	0.290
**School grade**						
High school (Ref.)	1		1		1	
Middle school	1.00 (0.87–1.15)	0.970	0.92 (0.77–1.10)	0.383	1.01 (0.81–1.26)	0.928
**Cigarette smoking**						
Never smoked (Ref.)	1		1		1	
Experimental	0.59 (0.52–0.68)	**<0.0001**	0.68 (0.57–0.82)	**<0.0001**	0.71 (0.58–0.89)	**0.002**
Established	0.27 (0.21–0.35)	**<0.0001**	0.16 (0.11–0.25)	**<0.0001**	0.35 (0.18–0.69)	**0.002**
**Other tobacco products use**						
Never used (Ref.)	1		1		1	
Ever used	0.39 (0.34–0.45)	**<0.0001**	0.45 (0.37–0.54)	**<0.0001**	0.47 (0.38–0.62)	**<0.0001**
**Family tobacco use**						
Does not use tobacco (Ref.)	1		1		1	
Use tobacco	0.72 (0.64–0.80)	**<0.0001**	0.79 (0.66–0.95)	**0.011**	0.81 (0.65–1.01)	0.066

AOR: adjusted odds ratio. The bold values are significant at p<0.05. Self-reported ‘Cigarette smoking risk perception’ assessed with the question: ‘How much do you think people harm themselves when they smoke cigarettes some days but not every day?’.

### Curiosity toward smoking among never smokers

Exposed adolescents were 42% more curious toward cigarettes (AOR=1.42; 95% CI: 1.09–1.50) compared with their not exposed peers. Curiosity was higher among exposed Hispanic adolescents (AOR=1.26; 95% CI: 1.10–1.44), and lower among Non-Hispanic Blacks (AOR=0.80; 95% CI: 0.66–0.96) compared to exposed Non-Hispanic Whites. Non-Hispanic Black adolescents who were not exposed to the anti-smoking campaign were 47% less likely to be curious (AOR= 0.53; 95% CI: 0.38–0.74) compared to their unexposed Non-Hispanic White peers.

Exposed youths who used other tobacco products had twice the cigarettes curiosity odds (AOR=1.96; 95% CI: 1.74–2.20) compared with those who did not consume tobacco. Cigarette curiosity was over two folds higher among the unexposed adolescents who were tobacco users (AOR=2.33; 95% CI: 1.87–2.89) compared with adolescents who did not use other tobacco products. Finally, exposed adolescents who had tobacco users in their household reported higher curiosity odds (AOR=1.37; 95% CI: 1.21–1.54) compared with the ones who did not live with tobacco users (Table 3).

**Table 3:** Logistic regression analysis of the factors associated with exposure to ‘The Real Cost’ anti-smoking campaign and curiosity about cigarettes among US adolescents aged 11–18 years who never smoked, NYTS 2018–2020

*Covariates*	*Exposed*		*Not exposed*		*Not Sure*	
	*AOR (95% CI)*	*p*	*AOR (95% CI)*	*p*	*AOR (95% CI)*	*p*
**Sex**						
Female (Ref.)	1		1		1	
Male	0.90 (0.82–0.99)	**0.037**	1.19 (0.98–1.45)	0.071	1.10 (0.54–0.94)	0.313
**Race/ethnicity**						
Non-Hispanic White (Ref.)	1		1		1	
Non-Hispanic Black	0.80 (0.66–0.96)	**0.019**	0.53 (0.38–0.74)	**<0.0001**	0.78 (0.53–1.14)	0.192
Hispanic	1.26 (1.10–1.44)	**0.001**	1.18 (0.97–1.45)	0.105	1.11 (0.89–1.40)	0.357
Non-Hispanic Asian	1.19 (0.92–1.54)	0.188	1.18 (0.86–1.63)	0.309	1.39 (0.85–2.27)	0.188
Non-Hispanic Other	1.05 (0.68–1.62)	0.814	0.75 (0.37–1.52)	0.426	1.19 (0.55–2.59)	0.654
**School grade**						
High school (Ref.)	1		1		1	
Middle school	1.54 (1.37–1.74)	**<0.0001**	1.23 (1.01–1.50)	**0.041**	1.57 (1.26–1.96)	**<0.0001**
**Other tobacco products use**						
Never used (Ref.)	1		1		1	
Ever used	1.96 (1.74–2.20)	**<0.0001**	2.33 (1.87–2.89)	**<0.0001**	2.00 (1.55–2.58)	**<0.0001**
**Family tobacco use**						
Does not use tobacco (Ref.)	1		1		1	
Use tobacco	1.37 (1.21–1.54)	**<0.0001**	1.24 (1.01–1.53)	**0.043**	1.59 (1.30–1.95)	**<0.0001**

AOR: adjusted odds ratio. The bold values are significant at p<0.05. Self-reported ‘Curiosity towards cigarette smoking’ (among never smokers) was assessed with the question: ‘Have you been curious about smoking cigarettes?’.

## DISCUSSION

To our knowledge, this is the first national study to explore the multi-year impact of the FDA’s ‘The Real Cost’ anti-smoking advertising campaign on cigarette risk perception and smoking curiosity among US adolescent population. We noted that the campaign has achieved high penetration levels, with most adolescents (63%) recalling exposure to at least one advertisement from the campaign, a finding consistent across demographic subgroups. Our report indicates that the FDA’s campaign has attained initial success when it comes to increasing risk perceptions among adolescents and that (high-risk) students reported high exposure levels to the campaign messages. On the other hand, a possible unintended consequence of the campaign was observed among exposed youths who never smoked cigarettes as they were found to have higher curiosity levels toward cigarette smoking.

While ‘The Real Cost’ campaign addressed youth relevant themes (physical appearance and loss of control), the overarching idea was that tobacco and smoking leads to adverse health effects, which may be expressed within the scope of risk perception^12^. Risk perception is often described as one’s perceived judgement on the probability or susceptibility to negative health outcomes^18^, and it is a necessary predictor in health behavior theories^19^. Evidence suggests that interventions that positively influence and alter risk perceptions could consequentially improve healthy behaviors. Thus, risk perceptions are often considered major indicators of campaign effectiveness in evaluations^18,20^. Mirroring previous reports, our study found that exposure to the campaign was associated with increased smoking risk perceptions linked to smoking cigarettes^12,21^. Youths who were positively exposed to the messages were more likely to have higher risk perceptions than unexposed ones^12,21^. Racial disparities were observed among Hispanic and Non-Hispanic Black adolescents, who appeared to be at the greatest disadvantage as it relates to perceiving cigarette smoking as risky when compared to their Non-Hispanic White peers. Moreover, exposed adolescents who never smoked cigarettes were more likely to perceive cigarettes as risky compared to experimental and established smokers. The disparity in risk perception was even wider among not-exposed youth who were established cigarette smokers compared to never smokers. Smokers could be underestimating health risks of cigarettes due to self-exempting beliefs, or cognitive dissonance-reducing beliefs, which are thoughts that one may hold that ‘exempt’ them from negative consequences^22^.

Adolescents who recalled exposure to the advertisements were evidently more curious about cigarettes than the ones who did not recall exposure to the anti-smoking campaign. Cigarettes curiosity levels were higher among exposed Hispanic youths and lower among Non-Hispanic Blacks compared to their Non-Hispanic White peers. Curiosity may indicate interest, and increased sensitivity to behaviorrelevant stimuli such as advertising, which could lead to impulsive behavior^5,23^. One possible explanation for this finding is that adolescents who were ‘committed never smokers’ might have been more receptive to remembering and recalling exposure to such advertisements. Moreover, public health communications need to compete for the public’s attention with several other compelling factors, such as previous industry marketing, established social norms, and addiction-driven behaviors^7^. Tobacco use among adolescents has been found to be associated with low perceived risks related to those behaviors^24^. A prominent explanation for young people’s smoking is that adolescents have poor decision-making and risk assessment skills, leading them to believe they are invulnerable to harm^25^. Therefore, public health anti-smoking campaigns are designed with an emphasis on ‘risk perception’ and ‘fear appeal’; the rationale behind this is that in ‘health communication’ , the audience needs to identify a risk before they can take positive steps toward health improvement^26^.

### Limitations

The current study has several limitations. The primary limitation is that the findings are restricted to associations because NYTS data are cross-sectional in nature and do not allow the measuring of pre-post ‘The Real Cost’ exposure changes, which challenges our ability to examine causal relationships. Additionally, NYTS data are collected from students in schools and therefore could not be generalizable to all adolescents in the US (i.e. homeschooled adolescents, school dropped outs, or youths detained in institutes). Moreover, this report involved self-reported data which may be subject to social desirability and recall bias. Further, due to data constraints, we could not empirically separate the individual mechanisms that would have explained the effects of media advertisements on normative beliefs (e.g. presumed influence, heuristic judgment).

Finally, the NYTS question used to assess awareness of the campaign did not ask about specific ads; this is not how awareness is assessed in traditional evaluations. Future longitudinal studies should be able to provide a clearer understanding of the ‘The Real Cost’ impact on smoking-related beliefs, attitudes and behaviors.

### Implications

The findings of this study hold some important implications for future public health campaigns design and implementation. Learning which campaign themes, and dissemination channels were associated with beliefs and attitudes for specific tobacco products, and specific high-risk populations, may inform future campaigns messaging strategy and media purchasing decisions. Further, these findings have many implications for emerging products, particularly those with a strong advertising component such as electronic cigarettes (e-cigarettes) which are currently the most commonly used tobacco product among youth.

## CONCLUSIONS

Our findings indicated that the FDA’s ‘The Real Cost’ anti-smoking campaign has achieved high penetration levels among US adolescent population. Exposure was associated with higher cigarette risk perceptions. On the other hand, students who never smoked cigarettes, were found to be more curious about smoking with exposure to the campaign advertisements. Therefore, future health communication plans should consider both the potential benefits and possible unintended consequences prior to launching such campaigns. Future research efforts should aim to explore the longitudinal impact of this campaign, particularly its effect on smoking related perceptions, attitudes and behavior. Additionally, researchers should consider studying the influence of the campaign on risk perception of emerging products among US youth, namely e-cigarettes, which are currently under the jurisdiction of the FDA.

## Data Availability

Data sharing is not applicable to this article as no new data were created. A (Public Use Dataset) was used for this study (https://www.cdc.gov/tobacco/data_statistics/surveys/nyts/data/index.html)
